# Factors influencing receptivity to future screening options for pancreatic cancer in those with and without pancreatic cancer family history

**DOI:** 10.1186/1897-4287-10-8

**Published:** 2012-06-27

**Authors:** Carmen Radecki Breitkopf, Pamela S Sinicrope, Kari G Rabe, Tabetha A Brockman, Christi A Patten, Robert R McWilliams, Shawna Ehlers, Gloria M Petersen

**Affiliations:** 1 Mayo Clinic College of Medicine, Department of Health Sciences Research, Charlton 6, 200 First Street SW, Rochester, MN, 55905, USA; 2 Mayo Clinic College of Medicine, Behavioral Health Research Program, 200 First Street SW, Rochester, MN, 55905, USA; 3 Mayo Clinic College of Medicine, Department of Psychiatry and Psychology, 200 First Street SW, Rochester, MN, 55905, USA; 4 Mayo Clinic College of Medicine, Department of Medical Oncology, 200 First Street SW, Rochester, MN, 55905, USA

**Keywords:** Pancreatic cancer, Health behavior, Perceived risk, Screening intentions

## Abstract

**Background:**

Pancreatic cancer (PC) is considered the most lethal cancer and approximately 10% of PC is hereditary. The purpose of the study was to assess attitudes of at-risk family members with two or more relatives affected with pancreas cancer (PC) toward PC risk and future screening options.

**Methods:**

At-risk family members and primary care controls were surveyed regarding perceived PC risk, PC worry/concern, attitude toward cancer screening, screening test accuracy, and intentions regarding PC screening via blood testing or more invasive endoscopic ultrasound (EUS).

**Results:**

PC family members reported greater perceived risk of PC than controls (54% vs. 6%, respectively, p < 0.0001). PC family members also reported higher levels of PC worry/concern than controls (p < 0.0001), although 19% of PC family members indicated they were “not at all concerned” about getting PC. PC family members indicated greater acceptance of a false-negative result on a PC screening test relative to controls (12% vs. 8%, p = 0.02). Both groups reported high (>89%) receptivity to the potential PC screening options presented, though receptivity was greater among PC family members as compared to controls (p < 0.0001) for EUS. In multivariable analyses, degree of PC concern (p < 0.0001) was associated with intention to screen for PC by blood test and EUS, while perceived PC risk was associated with likelihood of undergoing EUS only (p < 0.0001).

**Conclusions:**

Receptivity to screening options for PC appears high. Clinicians should address behavioral and genetic risk factors for PC and foster appropriate concern regarding PC risk among at-risk individuals.

## Introduction

Pancreatic cancer (PC) is the fourth leading cause of cancer death among men and women in the U.S. [1]. The incidence rates of PC have increased by 1.5% per year since 2004, and in 2012, it is estimated that there will be 43,920 new cases of PC and 37,390 deaths due to this disease [1]. The lifetime risk of PC is about 1 in 71 for males and females [1]. For all stages combined, the 5-year relative survival rate is 6% with survival at earlier stages being 22% [1]. The causes of this deadly disease are not well understood, but approximately 10% of pancreatic cancer is hereditary [2], and a person’s chance of developing this cancer increases two- to three-fold if a first-degree relative (parent, sibling or child) has PC [3]. Presently, the United States Preventive Services Task Force (USPSTF) recommends against routine screening for PC in the general population because of the low prevalence of this malignancy, the limited accuracy and invasiveness of the currently available tests, and the poor outcomes of treatment [4]. However, screening at-risk individuals is receiving increasing support [5-8] with a recommended threshold to offer screening to those who carry a ≥10-fold increased risk [9].

Recent advances in screening technology via serum or stool tests or endoscopic ultrasound (EUS) hold promise for detecting early-stage PC [10-15]. A blood or stool test for early detection of PC would be preferable to EUS because of lower invasiveness and cost, however, biomarkers for PC are elusive and the efficacy of emerging potential serum or stool panels remains unknown with regard to early detection. The ability of EUS to assist in diagnosing pancreatic malignancies has also been demonstrated [16-19]. However, concerns with EUS relate to its invasive nature, cost, accuracy and availability [16]. At the present time, blood and stool tests and EUS remain areas of research as potential screening tools for PC. Studies are underway to provide a stronger rationale for their use among appropriate groups at particular risk for developing PC [20,21], including a recent report addressing the psychological impact of PC surveillance among at-risk participants in a Dutch PC surveillance study [22]. This study, which included only at-risk individuals who already agreed to surveillance, demonstrated that surveillance was not associated with increased cancer worry or elevated anxiety or depression levels [22]. Understanding the perceptions of at-risk, unaffected PC family members who are not enrolled in surveillance regarding future screening options is important and comparing their perceptions to individuals not at particular risk of PC would fill an existing gap in the literature.

The objective of this study was to evaluate perceived PC risk, PC worry and concern, and receptivity to future PC screening options among at-risk unaffected family members of individuals with PC relative to individuals (controls) who more closely resemble the PC risk profile for the general population. Individuals with a family history of cancer may overestimate their personal cancer risk and report increased cancer-related worry or concern; these factors may in turn positively or negatively influence attitudes and behaviors toward screening among this higher-risk group [23-28]. Furthermore, this study sought to identify factors related to interest in screening for PC and to assess expectations surrounding the accuracy of PC screening tests.

## Materials and methods

### Study population and study procedures

All study procedures were approved by the Mayo Clinic Institutional Review Board and all subjects provided written informed consent to participate. Study subjects were at-risk unaffected family members enrolled in the Mayo Clinic Pancreatic Cancer Family Study, a study conducted as part of the Specialized Program of Research Excellence (SPORE) and Pancreatic Cancer Genetic Epidemiology Consortium studies [3,29] as well as primary care controls. At-risk family members included those with two or more first- or second-degree relatives affected with pancreatic adenocarcinoma. Control subjects included those attending Mayo Clinic for a general medical exam. Relatives of individuals with PC and controls comprising the comparison group for this study were recruited from 4/27/2004 -1/24/2008. All participants completed a survey addressing perceived PC risk, degree of PC worry/concern, attitude toward cancer screening in general, and intentions regarding uptake of PC screening if it were available as a blood test or endoscopic ultrasound (EUS).

### Measures

Demographic and medical history data were collected using a self-report survey instrument. The instrument included items assessing age, sex, race/ethnicity, education, smoking status (current, ever, never), household income, and number of family members affected by cancer.

Perceived risk of PC was assessed in absolute and comparative terms [30]. Specifically, the item “How likely do you think it is that you will get pancreatic cancer sometime in your life?” was used to assess absolute perceived risk. Response options on a 5-point scale ranged from “very likely” to “very unlikely.” Responses were grouped for analysis to reflect “likely” (including the responses “very likely” and “likely”) and “unlikely” (including the responses “no feeling or opinion,” “unlikely,” and “very unlikely”). Comparative risk perception was assessed by the item: “Compared to most people of your same age, sex, and race, what do you think your chances are of getting pancreatic cancer sometime in your life?” Response options included (1) “much higher” chance to (5) “much lower” chance.

Pancreatic cancer worry and concern was assessed by four items adapted from Lerman and colleagues [31]. Three items referred to the time period “during the past month” and queried: “How often have you thought about your chances of getting pancreatic cancer?” “How often have thoughts about your chances of getting pancreatic cancer affected your mood?” and “How often have thoughts about your chances of getting pancreatic cancer affected your ability to perform your daily activities?” Response options included “not at all or rarely,” “sometimes,” “often,” and “a lot”. A fourth item queried: “How concerned are you about getting pancreatic cancer?” to which participants responded using a 4-point Likert-type scale ranging from (1) “extremely concerned” to (4) “not at all concerned”. Cronbach’s alpha for these items was 0.78, demonstrating acceptable internal consistency. For multivariable analysis, responses were categorized as “concerned” (“extremely”/“moderately”) or “not concerned” (“mildly”/“not at all”) for ease of interpretation.

Participants’ willingness to take part in PC screening if it were a blood test or EUS was assessed by two items, using a four-point Likert response format with responses ranging from “very unlikely” to “very likely”. The questions read, “If a blood test were available to screen for pancreatic cancer, how likely is it that you would take it?” and “If a test were available to screen for pancreatic cancer that required an upper endoscopic examination (you would be sedated, or made drowsy, and a flexible tube would be inserted through your mouth into your stomach), how likely is it that you would take it?”

As data are not yet available regarding actual false-negative or false-positive rates for PC screening tests and the concepts themselves can be difficult to understand [32], expectations surrounding the accuracy of a PC screening test were assessed more generally. The following three items were presented: “It would be OK if a test detects less than 100% of people who have pancreatic cancer,” “If a test says I do NOT have pancreatic cancer when I really DO, that is OK with me,” (false-negative) and “If a test says I may have pancreatic cancer when I really do NOT, that is OK with me” (false-positive). A fourth item addressed cancer screening more generally: “It would be OK if a test says cancer may be present in a person who really does NOT have cancer.” These four items were assessed using a “strongly agree” to “strongly disagree” rating scale with a “do not know” option.

### Statistical analysis

Data were descriptively summarized using frequencies and percentages for categorical variables, and means and standard deviations for continuous variables. Comparisons between group (PC family members or controls) and psychosocial characteristics, likelihood to undergo screening, and expectations of screening accuracy were evaluated using multivariable logistic regression adjusting for sex, age, smoking, and family history (first degree relatives) of any cancer; odds ratios and 95% confidence intervals (CI) are presented. Because multiple members from the same family could participate in the PC family member group, analyses were also conducted which accounted for possible non-independence using generalized estimating equation (GEE) methodology. Family-specific correlations for screening via blood test and EUS were modeled using an exchangeable covariance matrix. All statistical analyses were performed using SAS 9.2 (SAS Institute Inc., Cary, NC).

## Results

### Sample characteristics

A total of 378 PC family members and 1528 controls consented and were sent a survey; 361 (96%) PC family members (representing 115 different families) and 1045 (68%) controls completed the survey. Mean ages in the two groups were 53.7 (±14.6) and 65.6 (±10.7) years, respectively (p < 0.001). Relative to controls, PC family members were younger, more likely to be female, current smokers, and have more first degree relatives with any cancer (Table 1).

**Table 1:** Participant Demographics by Group (n = 1406)

	**Control (n = 1045)**	**PC Family Member (n = 361)**	**P**
	**n (%)**	**n (%)**	
Sex			0.0004
Male	512 (49)	138 (38)	
Female	533 (51)	223 (62)	
Age (y)			<0.0001
Mean (±SD)	65.6 (10.7)	53.7 (14.6)	
<30	5 (0.5)	23 (6)	
30-49	91 (9)	117 (32)	
50-64	329 (31)	131 (36)	
65-79	557 (53)	79 (22)	
80+	63 (6)	11 (3)	
Current Smoker			<0.0001
Yes	35 (3)	41 (11)	
No	1002 (97)	320 (89)	
Ever Smoker			0.17
Yes	468 (45)	178 (49)	
No	569 (55)	183 (51)	
Education			0.06
High school or less	317 (31)	91 (25)	
Greater than high school	722 (69)	269 (75)	
Household income ($)			0.71
Less than 20,000	91 (5)	23 (6)	
20,000- 35,000	128 (13)	44 (13)	
>35,000- 50,000	164 (17)	52 (15)	
>50,000- 75,000	204 (21)	70 (21)	
>75,000	415 (44)	151 (45)	
Proportion of FDR with any cancer, % (range)	12 (0–100)	17 (0–100)	<0.0001

### Perceived pancreatic cancer risk

A majority (54%) of PC family members reported that they were likely to get PC in their lifetime relative to 6% of controls (p < 0.0001). A similar pattern was observed for comparative risk estimates, i.e., perceptions of risk relative to most people of the same age, sex, and race, with a majority (67%) of PC family members reporting a greater risk vs. 5% of controls (p < 0.0001) (Table 2).

**Table 2:** Psychosocial Characteristics and Outcome Variables by Group

	**Control (n = 1045) N (%)**	**PC Family Member (n = 361) N (%)**	**P**
PSYCHOSOCIAL CHARACTERISTICS			
Chances of getting PC sometime in life compared to most people of the same age, race and sex			<0.0001^1^
Much higher	14 (1)	130 (37)	
Higher	40 (4)	108 (30)	
About the same	461 (46)	93 (26)	
Lower	186 (18)	7 (2)	
Much lower	308 (31)	17 (5)	
Likelihood of getting pancreatic cancer sometime in life			<0.0001
Likely	61 (6)	193 (54)	
Not likely	953 (94)	163 (46)	
Frequency of thoughts about chances of getting PC during past month			<0.0001^2^
Not at all or rarely	975 (95)	190 (53)	
Sometimes	42 (4)	120 (34)	
Often	3 (<1)	30 (8)	
A lot	2 (<1)	18 (5)	
Frequency of thoughts about chances of getting PC affecting mood during the past month			<0.0001^2^
Not at all or rarely	1004 (99)	294 (83)	
Sometimes	13 (1)	48 (14)	
Often	0 (0)	7 (2)	
A lot	1 (<1)	6 (2)	
Frequency of thoughts about chances of getting PC affecting ability to perform daily activities during the past month			0.001^2^
Not at all or rarely	1008 (99)	340 (96)	
Sometimes	10 (1)	12 (3)	
Often	0 (0)	1 (<1)	
A lot	0 (0)	3 (<1)	
Degree of concern about getting PC			<0.0001^3^
Extremely Concerned	16 (2)	37 (10)	
Moderately Concerned	65 (6)	109 (31)	
Mildly Concerned	334 (33)	143 (40)	
Not at all concerned	607 (59)	68 (19)	
OUTCOME VARIABLES			
Likelihood of undergoing PC screening if it were a blood test			0.08^4^
Extremely Likely	452 (44)	224 (63)	
Likely	465 (45)	113 (32)	
Unlikely	81 (8)	19 (5)	
Extremely Unlikely	25 (2)	1 (<1)	
Likelihood of undergoing PC screening if it were EUS			<0.0001^4^
Extremely Likely	163 (16)	145 (41)	
Likely	390 (39)	121 (34)	
Unlikely	342 (34)	78 (22)	
Extremely Unlikely	116 (11)	12 (3)	

^1^ P-value obtained from combining “Much higher” and “Higher” versus “About the same”, “Lower”, and “Much lower”.

^2^ P-value obtained from combining “Sometimes”, “Often”, “A lot” versus “Not at all or rarely”.

^3^ P-value obtained from combining “Extremely Concerned”, “Moderately Concerned” and “Mildly Concerned” versus “Not at all concerned”.

^4^ P-value obtained from combining “Extremely Likely” and “Likely” versus “Unlikely” or “Extremely Unlikely”.

### Pancreatic cancer related worry/concern

PC family members reported more frequent thoughts in the past month about getting PC (47% vs. 5%, respectively, p < 0.0001), with these thoughts affecting mood. Overall, 81% of PC family members reported some degree of concern (“extreme,” “moderate,” or “mild”) about getting PC relative to 41% of controls, (p < 0.0001) (Table 2).

### Expectations surrounding screening test accuracy

A majority (>75%) of both PC family members and controls expressed acceptance of a PC screening test that was not 100% accurate (Table 3). PC family members indicated greater acceptance of a false-negative result on a PC screening test relative to controls (12% vs. 8%, p = 0.002). Acceptance rates for false-positive test results were slightly higher overall than acceptance rates for false-negatives; however no differences were observed between the two groups with regard to acceptance of false-positive results both for a cancer screening test in general, and a screening test for PC (both p > 0.05; Table 3).

**Table 3:** Expectations of Screening Accuracy by Group

	**Controls (n = 1045) N (%)**	**PC Family Members (n = 361) N (%)**	**P-value**^1^
**Global View**			
It would be OK if a test detects less than 100% of people who have pancreatic cancer.			0.27
Strongly Agree	401 (40)	133 (38)	
Somewhat Agree	360 (36)	143 (41)	
Somewhat Disagree	74 (7)	33 (9)	
Strongly Disagree	90 (9)	18 (5)	
Do not know	80 (8)	21 (6)	
It would be OK if a test says cancer may be present in a person who really does NOT have cancer. (“false-positive”)			0.37
Strongly Agree	81 (8)	27 (8)	
Somewhat Agree	206 (20)	80 (22)	
Somewhat Disagree	217 (22)	75 (21)	
Strongly Disagree	437 (43)	161 (45)	
Do not know	67 (7)	15 (4)	
**Personal View**			
If a test says I do NOT have pancreatic cancer when I really DO, that is OK with me. (“false-negative”)			0.02
Strongly Agree	34 (3)	17 (5)	
Somewhat Agree	47 (5)	25 (7)	
Somewhat Disagree	112 (11)	57 (16)	
Strongly Disagree	762 (76)	251 (70)	
Do not know	53 (5)	7 (2)	
If a test says I may have pancreatic cancer when I really do NOT, that is OK with me. (“false-positive”)			0.08
Strongly Agree	64 (6)	25 (7)	
Somewhat Agree	146 (14)	63 (18)	
Somewhat Disagree	219 (22)	77 (22)	
Strongly Disagree	510 (51)	181 (51)	
Do not know	71 (7)	12 (3)	

^1^ P-value obtained from combining “Strongly Agree” and “Somewhat Agree” versus “Somewhat Disagree” and “Strongly Disagree”. “Do not know” responses were excluded.

### Likelihood of undergoing PC screening

Overall, 89% of controls and 95% of PC family members reported that they were “likely” or “extremely likely” to undergo PC screening via a blood test (p = 0.08). Although these rates were comparatively lower for PC screening via EUS, (55% of controls and 75% of PC family members), likelihood of uptake was higher in the PC family member group relative to controls (p < 0.0001). Those who were “likely” vs. “not likely” to undergo PC screening via a blood test differed by group (PC family member vs. control) (p = 0.007), age (p = .001), perceived PC risk (p < 0.0001), and degree of cancer worry/concern (p < 0.0001) in univariable analyses (Table 4). In a multivariable logistic regression model, the likelihood of screening for PC via a blood test was associated with greater degree of cancer worry/concern (p < 0.0001). Group, age, and perceived PC risk were no longer independently associated with screening likelihood by blood test.

**Table 4:** Intention to Screen for Pancreatic Cancer (PC): Blood Test Univariable and Multivariable Models

	**Not Likely N (%)**	**Likely N (%)**	**Univariable**	**Multivariable**	
			**p-value**	**Odds ratio (95% CI)**	**p-value**
N	126	1254			
Group			0.007	0.7 (0.4, 1.3)	0.25
Control	106 (84)	917 (73)			
PC Family member	20 (16)	337 (27)			
Sex			0.31	1.3 (0.9, 2.0)	0.17
Male	53 (42)	587 (47)			
Female	73 (56)	667 (53)			
Age (y), mean ± SD	66.2 ±13.4	62.1 ±12.8	0.001	0.98 (0.97, 1.00)	0.07
Likelihood of getting PC sometime in your life			<0.0001	2.5 (0.9, 6.9)	0.08
Likely	6 (5)	247 (20)			
Not likely	118 (95)	985 (80)			
Degree of concern about getting PC			<0.0001	5.0 (3.0, 8.5)	<0.0001
Concerned	20 (16)	682 (55)			
Not concerned	105 (84)	566 (45)			

Similar analyses were conducted with regard to the likelihood of undergoing screening via EUS. In univariable analyses, likelihood of screening via EUS was associated with the same factors as screening via a blood test: group (p < 0.0001), age (p = 0.002), perceived PC risk (p < 0.0001), and degree of cancer worry/concern (p < 0.0001) (Table 5). In the multivariable context, only perceived PC risk (p < 0.0001) and degree of cancer worry/concern (p < 0.0001) remained significant. Analyses accounting for the possibility that data are correlated within a family did not appreciably change results in any of the models (results not shown).

**Table 5:** Intention to Screen for Pancreatic Cancer (PC): Endoscopic Ultrasound (EUS) Univariable and Multivariable Models

	**Not Likely N (%)**	**Likely N (%)**	**Univariable**	**Multivariable**	
			**p-value**	**Odds ratio (95% CI)**	**p-value**
N	548	819			
Group			<0.0001	1.2 (0.9, 1.7)	0.30
Control	458 (84)	553 (68)			
PC Family member	90 (16)	266 (32)			
Sex			0.82	1.1 (0.9, 1.4)	0.46
Male	257 (47)	379 (46)			
Female	291 (53)	440 (54)			
Age (y), mean ± SD	63.8 ± 12.4	61.6 ± 13.2	0.002	1.002 (0.99, 1.01)	0.63
Likelihood of getting PC sometime in your life			<0.0001	3.4 (2.2, 5.3)	<0.0001
Likely	36 (7)	214 (27)			
Not likely	505 (93)	589 (73)			
Degree of concern about getting PC			<0.0001	2.1 (1.6, 2.6)	<0.0001
Concerned	192 (35)	502 (62)			
Not concerned	354 (65)	313 (38)			

## Discussion

Comprehensive efforts are underway to better understand the etiology of PC, to improve treatment outcomes, and to develop and evaluate effective screening technologies [8,33]. Receptivity toward potential future screening options for pancreatic cancer among those at significant risk is an important, yet understudied area. Overall, receptivity toward screening was higher among PC family members relative to controls; of the potential screening options studied, receptivity was greater for the less invasive method (blood test vs. EUS). These findings are consistent with qualitative data reported by Lewis [34] and are important with regard to developing early detection methods that will be acceptable to the individuals for whom they are intended. Previous studies including highly-selected, at-risk individuals have reported actual uptake of PC screening ranging between 61% (for EUS) and 67% (for MRI), which reflects variation in individual preferences and provides evidence that uptake cannot always be assumed [6,8]. Moreover, this study established that individuals who have family members with PC may exhibit greater worry and concern about PC, perceive greater PC risk for themselves and that *these psychological responses are independent, positive predictors of intention to undergo screening* for PC via blood test (worry/concern) and EUS (worry/concern and perceived risk) were such screening tests to become available in the future.

These findings are consistent with theoretical models of voluntary health behavior that include the construct of perceived vulnerability, such as protection motivation theory [35] and the health belief model [36,37]. Specifically, these models posit that willingness to undergo invasive procedures would be less likely for an average risk group of individuals, while those with heightened risk perception, greater perceived susceptibility or vulnerability and increased awareness of the severity of the target disease, would express greater likelihood of undergoing even an invasive screening test such as EUS. In this investigation, it was these psychological constructs that were predictive of likelihood of screening in the multivariable context, not group status, suggesting that at-risk individuals (PC family members) who fail to recognize their vulnerability may be no more likely than average-risk individuals (controls) to undergo screening. This point underscores the importance of understanding the nature of risk perception and concern in cancer screening and the importance of allowing such perceptions to appropriately drive screening behavior. It also underscores the importance of appropriate education on risk to family members. In this study, individuals who perceived themselves as likely to get PC in their lifetime were over three times as likely as those who did not hold this perception to be willing to undergo EUS screening, irrespective of group status. Thus, there is a great need to measure and understand the complexities of these constructs more fully in future research on PC, perhaps using multi-dimensional measures of risk beliefs such as the one developed by Hay and colleagues [38,39].

In this study, PC family members perceived their risk of developing PC during their lifetime as greater compared to others of their same age, sex, and race. They also reported significantly higher levels of PC-related thoughts and concerns. These findings are consistent with the literature on perceived risk and concern among those at increased risk of cancer due to family history. However, previous studies have focused primarily on breast, colorectal, and lung cancer [32,40-42]. PC is different from these cancers in that there are currently no recommended screening options, and, while there are lifestyle-related factors that affect PC risk such as obesity [43] and cigarette smoking [44], no single factor has been identified that can dramatically reduce risk. Given the current limitations of the science of PC prevention, methods of early detection are needed.

Interestingly in this study, nearly 1 in 5 PC family members indicated they were “not at all worried” about getting PC. This finding could reflect lack of awareness regarding familial risk, a general belief that worry is unproductive, illusion of unique invulnerability [45,46] or a sense of futility in worrying about this deadly disease based on experience with a family member. Each of these possible explanations warrants empirical study, as lack of worry/concern could lead to complacency among a group of individuals who may benefit greatly from vigilance regarding early warning signs or heightened awareness of developments in screening. Patient education regarding PC risk and recommendations for surveillance among those at high risk is needed [34].

Sensitivity and specificity of screening tests are sophisticated, yet critical concepts for patients to understand prior to undergoing a screening test. The difference observed in this study between PC family members and controls with regard to acceptance of false negatives is a novel finding warranting further study. Greater acceptance of personal false negative test results among PC family members may reflect an experiential bias (relative to controls) that treatment is only rarely successful in changing the course of the disease, thus, “missing” the diagnosis can be viewed with greater acceptance. This interpretation, if valid, may reflect both cognitive (actual knowledge-based) and affective (emotional) responses resulting from experience with the course of PC in their family member. This same logic would support the trend-level findings observed regarding greater global acceptance of missing a diagnosis of PC among PC family members as compared to controls.

This research has several strengths including its focus on cancer of the pancreas, which remains the deadliest cancer. In addition, this research is one of the few psychosocial studies involving a relatively large sample of PC family members that also includes a control group. Finally, the importance and timeliness of the research cannot be understated; the incidence of PC is continuing to rise and effective screening methods are the focus of ongoing translational research. Despite these strengths, several limitations are important to note including non-independence that was unique to the PC family sample. Statistical approaches such as GEE can account for non-independence, but our findings should still be interpreted with caution. Moreover, PC families with BRCA1/2 and CDKN2A mutations were not excluded from the study; these families may have greater familiarity with cancer screening tests and their attitudes may differ in important ways from other PC family members. Second, this was a cross-sectional study assessing behavioral intentions toward hypothetical screening tests; it is inappropriate and premature to extrapolate these findings to future participation in screening tests for pancreatic cancer. Nonetheless, the utility of assessing intentions and acceptability regarding new methods of early cancer detection and prevention prior to their availability has been demonstrated in other cancers (e.g., spiral CT for early detection of lung cancer [32] and HPV vaccination for the prevention of cervical cancer [47]); such research is critical for the successful development and uptake of new strategies to detect and prevent cancer. Finally, as one of the first studies to provide data about receptivity to screening options for PC, it is limited in scope and not comprehensive. The assessments of perceived risk and acceptability of false-negatives/false-positives in this study avoided numeracy concerns but were compromised by imprecision. Future studies should explore concerns related to sensitivity and specificity of potential screening tests in greater detail as well as other barriers to early detection such as cost and availability; whether screening confers actual improvement in survival for PC remains to be determined. Addressing each of these issues in future studies of individuals and families at significant risk of pancreatic cancer will facilitate collective efforts toward the development and implementation of appropriate and acceptable screening methods targeting high-risk individuals.

## Competing interests

The author(s) declare that they have no competing interests.

## Authors’ contributions

CRB and PSS drafted the manuscript. GMP and CRB made substantial contributions to the analysis and interpretation of the data. KGR performed the statistical analysis. PSS, CAP, RRM, SE, and GMP conceived of the study, participated in its design, and constructed the survey. TAB coordinated the research effort and helped to revise the manuscript. GMP provided critical intellectual content and expertise from the conception to the conclusion of this research. All authors read and approved the final manuscript.
